# Effect of Gender on the Outcome of Patients Receiving Immune Checkpoint Inhibitors for Advanced Cancer: A Systematic Review and Meta-Analysis of Phase III Randomized Clinical Trials

**DOI:** 10.3390/jcm7120542

**Published:** 2018-12-12

**Authors:** Antonino Grassadonia, Isabella Sperduti, Patrizia Vici, Laura Iezzi, Davide Brocco, Teresa Gamucci, Laura Pizzuti, Marcello Maugeri-Saccà, Paolo Marchetti, Gaetana Cognetti, Michele De Tursi, Clara Natoli, Maddalena Barba, Nicola Tinari

**Affiliations:** 1 Department of Medical, Oral and Biotechnological Sciences and CeSI-MeT, G. D’Annunzio University, 66100 Chieti, Italy; laura.iezzi@unich.it (L.I.); davideabilly@gmail.com (D.B.); michele.detursi@unich.it (M.D.T.); natoli@unich.it (C.N.); ntinari@unich.it (N.T.); 2 Department of Bio-Statistics, RCCS Regina Elena National Cancer Institute, 00144 Rome, Italy; isabella.sperduti@ifo.gov.it; 3 Division of Medical Oncology 2, IRCCS Regina Elena National Cancer Institute, 00144 Rome, Italy; patrizia.vici@ifo.gov.it (P.V.); laura.pizzuti@ifo.gov.it (L.P.); marcello.maugerisacca@ifo.gov.it (M.M.-S.); maddalena.barba@ifo.gov.it (M.B.); 4 Medical Oncology, Sandro Pertini Hospital, 00157 Rome, Italy; t.gamucci@libero.it; 5 Scientific Direction, Regina Elena National Cancer Institute, 00144 Rome, Italy; 6 Oncology Unit, Department of Clinical and Molecular Medicine, Medical Oncology, Sapienza University, 00185 Rome, Italy; paolo.marchetti@uniroma1.it; 7 Digital library, Knowledge Center “Riccardo Maceratini” and Patient Library, Regina Elena National Cancer Institute, 00144 Rome, Italy; gaetana.cognetti@ifo.gov.it

**Keywords:** immune checkpoint inhibitors, anti-PD-1/PDL-1, anti-CTLA-4, gender, sex, nivolumab, pembrolizumab, atezolizumab, ipilimumab, durvalumab

## Abstract

Evidence has recently emerged on the influence of gender on the immune system. In this systematic review and meta-analysis of phase III randomized clinical trials (RCTs), we explored the impact of gender on survival in patients with advanced cancer treated with immune checkpoint inhibitors (ICIs). We performed a comprehensive search of the literature updated to April 2018, including the Cochrane Central Register of Controlled Trials, PubMed, and EMBASE. We extracted data on study characteristics and risk of bias in duplicate. Of 423 unique citations, 21 RCTs were included, inherently to 12,635 patients. Both males and females showed reduced risk of death associated with ICIs use (HR 0.73, *p* < 0.001 and HR 0.77, *p* < 0.001, respectively). Subgroup analyses by specific ICI showed similar OS in both genders for anti-PD-1/PDL-1. Anti-CTLA-4 use was associated with longer OS in men only (HR 0.77, *p* < 0.012), with the exception of melanoma (in women, HR 0.80, *p* = 0.006). PFS was longer in men than in women (HR 0.67, *p* < 0.001 and HR 0.77, *p* = 0.100, respectively). Conclusively, ICIs use was associated with more favorable outcomes in men, particularly for anti-CTLA-4 agents. In melanoma, not gender-related factors may influence the anti-tumor immune response evoked by ICIs.

## 1. Introduction

It is increasingly recognized that gender-related differences affect health status and impact on relevant outcomes in chronic pathologic conditions spanning from cardiovascular diseases to cancers [1]. Indeed, compared to men, women experience more frequently heart failure or strokes as complications of hypertension and atrial fibrillation, respectively [2]. In addition, mortality for acute myocardial infarction is higher in women than in men, while men have a higher risk of ischemic sudden death [3].

Similarly, when excluding sex-specific tumors such as ovarian and prostate cancers, several cancer types occur differently in men and women, displaying different behavior in terms of disease progression, response to treatment and prognosis [4,5]. The incidence and mortality of colorectal cancer are higher in women than in men, presumably for a higher percentage of right-sided colon cancer and more aggressive molecular features such as BRAF mutation and microsatellite instability [6]. Similar examples include bladder cancer, which is more common in men but has a worse prognosis in women; urothelial carcinoma and renal cell carcinoma, which are more frequent and associated with unfavorable prognosis in men; and melanoma, which is associated with better survival in women [7,8].

Although molecular differences in cancer cells have been described between men and women [9], the existence of a gender-dependent disparity in immune response may play a major role in influencing tumor outcome [10]. Compared to men, women tend to trigger and sustain a stronger immune response against infections [11] and show an increased propensity to develop autoimmune diseases [12]. Both innate and adaptive immune responses are higher in women than in men. In more detail, women exhibit higher efficiency of the antigen presenting cells (APCs) and macrophage activation, and higher levels of B cells, antibody production, CD4+ T cells, CD4/CD8 ratio, and T helper (Th) 2 cell response, while men have higher levels of CD8+ T cells, regulatory T (Treg) cells, and Th1 cell response [13]. Sexual immune dimorphism has been related to differences in terms of (i) expression of chromosome X-linked immune-related genes such as TLR7, TLR8, IL-2, IL-4, IL-15, FOXP3 [14,15]; (ii) hormonal modulation of immune response by estrogen, progesterone and testosterone [16,17]; (iii) influence of gut microbiome on immune competency [18,19]. 

Over the past two decades some molecular mechanisms implicated in cancer development and progression have been elucidated, including angiogenesis, aberrant receptor tyrosine kinase (RTK) activation, and loss of function of enzymes involved in DNA damage response and repair. From this knowledge there has been a veritable upsurge of several therapeutic interventions, in particular anti-angiogenetic agents [20], tyrosin-kinase inhibitors [21] and DNA damage and repair inhibitors [22,23] that have positively impacted cancer outcomes.

In recent years, the inhibition of critical pathways involved in T cell suppression, achieved by treatment with monoclonal antibodies against CTLA-4, PD-1 or PDL-1, the so called immune checkpoint inhibitors (ICIs), demonstrated a long-lasting response in several types of cancer and has become a standard of treatment for melanoma, non small-cell lung cancer (NSCLC), and Renal Cell Carcinoma (RCC) [24]. Given the considerable gender-related differences in immune response, it is conceivable that a diverse anti-tumor effect of ICIs in men and women may be observed. This has recently fostered the conduct of two systematic reviews and meta-analyses and one meta-analysis on treatment outcomes in cancer patients treated with ICIs in randomized clinical trials (RCTs). Overall, the evidence observed supports more favorable outcomes in men than in women [25,26,27].

We now add to the previously mentioned works by proposing a systematic review and meta-analysis which includes more recently published studies. Most importantly, as pointed out in the methods section and discussion, we exclusively focused on phase III RCTs of ICIs efficacy in advanced cancer patients, whereas both phase II and III RCTs were included in the previously cited works.

## 2. Materials and Methods

### 2.1. Search Strategy

We performed a systematic search in PubMed, EMBASE, and Cochrane Central Register of Controlled Trials (CENTRAL), up to April 2018, to identify phase III, RCTs comparing ICIs (anti-CTLA-4, anti-PD1, or anti-PD-L1) versus standard treatment or placebo, and reporting clinical outcomes of OS and/or PFS by gender. No language restrictions were applied. The PubMed’s “related articles” feature was used to identify further papers. The reference lists of the studies included were also screened. An expert librarian was involved in the design of the search strategy and in the conduct of the literature search. Accordingly, we searched publications using the following text keywords: Nivolumab, Pembrolizumab, Atezolizumab, Durvalumab, Ipilimumab, Avelumab, PDR001, Lambrolizumab, Tremelimumab, Checkpoint inhib*, anti-PD1, anti-PDL1, randomized controlled trial, controlled clinical trial, randomized, placebo, phase 3, phase III, RCT clinical trials, randomly, trial. We excluded from the search the following keywords: review, meta-analysis, phase 1, phase I, phase 1b, phase II, phase 2, phase 2b, case report, quality of life, FDA approval, guidelines, and real-world.

Exclusion criteria were the following: trials on breast, ovarian, prostate and testicular cancer, phase I and phase II trials, trials in which all arms received ICIs, and duplicates of already included studies. If multiple publications of the same trial were retrieved, only the first publication was included. 

### 2.2. Data Extraction

Two reviewers independently screened titles and abstracts for eligibility and subsequently extracted data using pilot-tested, ad hoc forms. Disagreements were solved by discussion or consultation with a third reviewer. The data extracted related to participants, intervention and outcomes of interest.

From each single RCT, selected according to the aforementioned criteria, the following data were extracted: treatment setting and regimens, total number of patients randomized to each study arm, number of patients treated in each study arm, control arm including/not including placebo, co-interventions, total number of female and male patients, total number of progressions and deaths by gender, gender-stratified Hazard Ratios (HR) and 95% confidence intervals (CIs) for OS and/or PFS, and whether or not the trial noted a statistically significant difference in survival between the compared arms. No data were provided by the studies regarding age distribution by gender. The lack of comparable age groups in men and women did not allow considering gender-related age as variable in the meta-analysis. 

### 2.3. Statistical Analysis

Risk of bias was assessed at the study level for each of the RCTs included in full agreement with the Cochrane Collaboration’s “Risk of bias” tool [28]. Two review authors independently assessed the methodological quality based on: Sequence generation; Allocation concealment; Blinding of patients and personnel; Blinding of outcome assessors; Incomplete outcome data; Selective outcome reporting; ITT analysis; Additional sources of bias. 

We compared treatments using Hazard Ratio and 95% confidence intervals. Heterogeneity was evaluated by *X*^2^ *Q* test and *I*^2^ statistic [29]. For the Q test, *p* < 0.05 indicated significant heterogeneity; for the *I*^2^ statistics, an *I*^2^ value >50% was considered significant. The pooled Hazard Ratio (HR) estimate was calculated using a random-effect model [30]. Our results are graphically displayed as forest plots, with HR < 1.0 indicating better outcome in the experimental arm. Substantial heterogeneity was explored in subgroup analyses by type of ICIs (anti-PD1/PDL-1 or anti-CTLA-4) and type of tumor (Melanoma or NSCLC). Publication bias was evaluated by visual inspection of funnel plots. Calculations were accomplished using the Comprehensive Meta-Analysis Software, version v.2.0 (CMA, Biostat, Englewood, NJ, USA).

## 3. Results

### 3.1. Study Description

**Figure 1:**
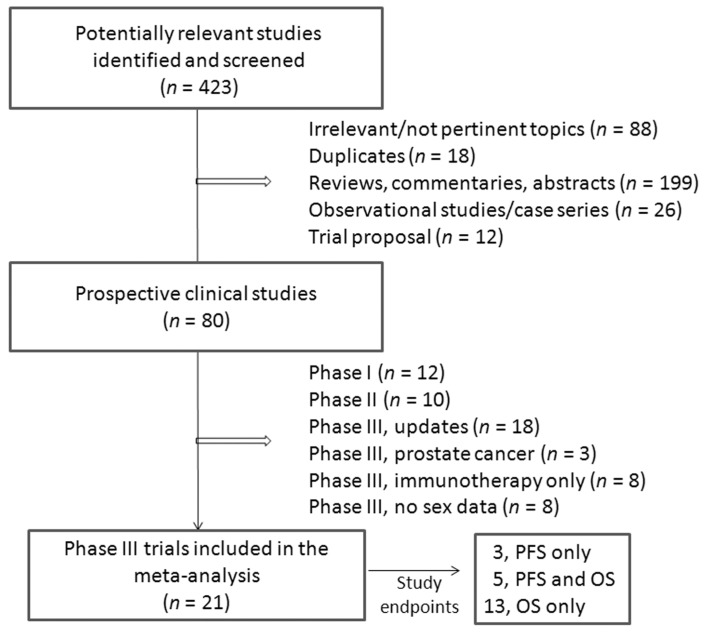
Flow diagram of study selection.

The flow diagram of the study selection process is shown in Figure 1. We identified 423 articles reporting on the use of ICIs for solid cancer treatment. Among them, 343 were excluded based on the title or abstract screening (88 irrelevant topics, 18 duplicates, 199 reviews/commentaries/abstracts, 18 observational studies, 12 proposals) or as a result of full text screening (8 observational studies). Of the remaining 80 studies, 22 were excluded because phase I or phase II studies, 18 because representing an update of previously published studies, 3 were studies on prostate cancer, 8 for the lack of appropriate control arms, and 8 for the lack of gender stratification. 

Twenty-one studies met the selection criteria and were included in the analysis (Table 1) [31,32,33,34,35,36,37,38,39,40,41,42,43,44,45,46,47,48,49,50,51]. One of them had two experimental arms, one with Ipilimumab (A) and one with Ipilimumab plus Gp100 (B) [31]. Both arms were separately included in the analysis. Another study was planned with two experimental arms using Pembrolizumab at 2 different doses (2 mg and 10 mg) [37]. In this case, a pooled analysis was considered.

Patients were mostly affected by metastatic melanoma (5 studies) [31,32,33,37,47] or non-small cell lung cancer (NSCLC) (10 studies) [34,35,38,40,42,44,46,48,50,51]. Other studies included renal cell carcinoma (RCC) [36,49], small-cell lung carcinoma (SCLC) [39], head and neck cancer [41], urothelial carcinoma [43], and gastric cancer [45]. Overall, 12,635 patients were included in our meta-analysis, 8410 males and 4225 females. Of them, 11,318 (7519 men and 3799 women) provided data for OS and 3746 (2384 men and 1362 women) for PFS. 

### 3.2. Risk of Bias Assessment

Results from the assessment of risk of bias are summarized in Table S1 (supplementary material). Overall, the methodological quality of the RCTs included was judged as acceptable. 

### 3.3. Effect of Sex on Overall Survival

**Figure 2:**
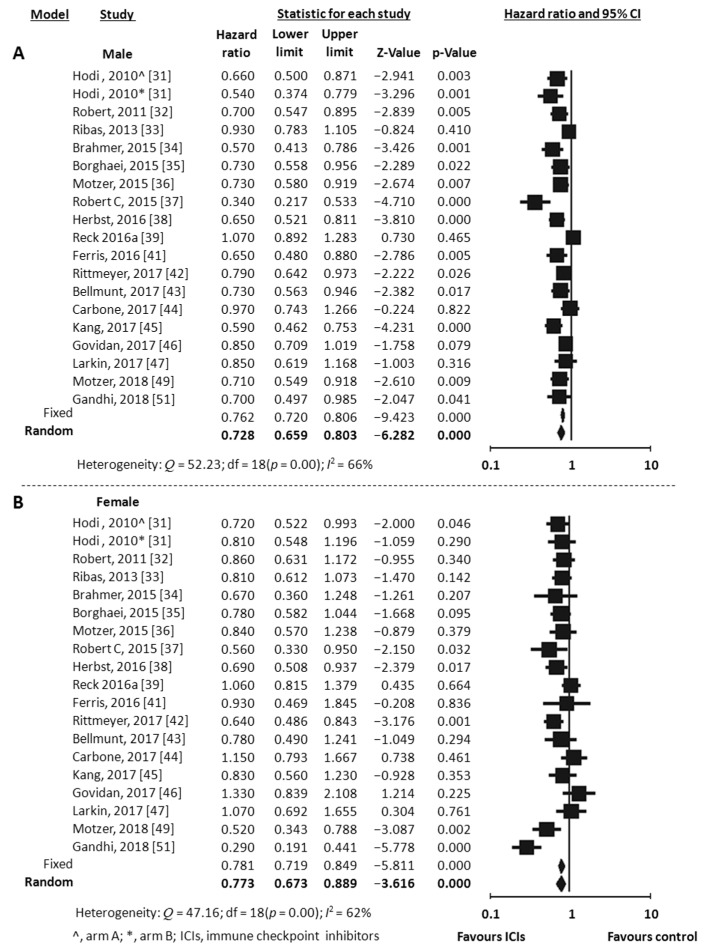
Meta-analysis results for OS with immune checkpoint inhibitors (ICIs). (**A**), male; (**B**), female.

**Figure 3:**
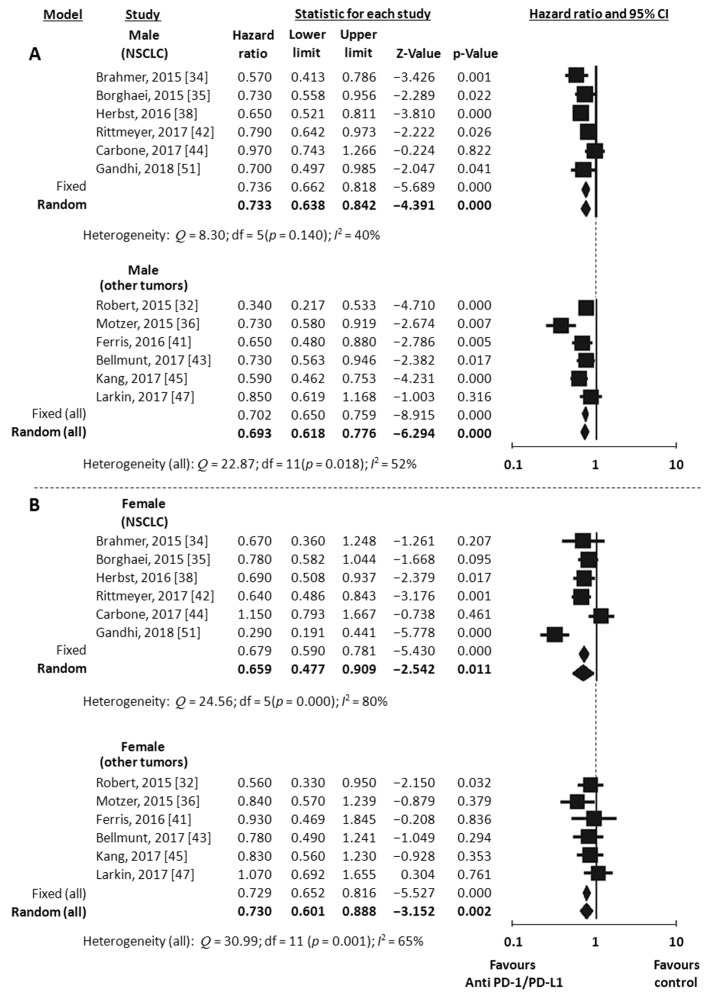
Meta-analysis results for OS with anti-PD-1/PDL-1. Studies on NSCLC were separately analyzed. (All) refers to the results of the analysis performed on the entire population (12 studies). NSCLC, non small-cell lung cancer; (**A**), male; (**B**), female.

**Figure 4:**
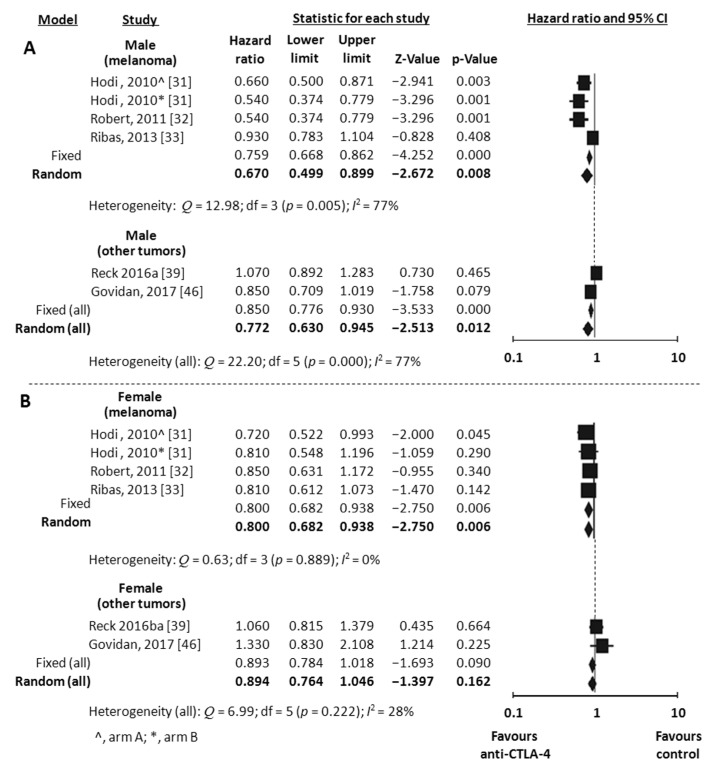
Meta-analysis results for OS with anti-CTLA-4. Studies on melanoma were separately analyzed. (All) refers to the results of the analysis performed on the entire population (6 studies). (**A**), male; (**B**), female.

Overall Survival (OS) data stratified by gender were available in 18 studies, including the one with 2 experimental arms [31,32,33,34,35,36,37,38,39,41,42,43,44,45,46,47,49,51]. Considering the random-effects model, both males and females showed a significant reduced risk of death when treated with ICIs compared to control. The HR was 0.73 for men (95% CI 0.66–0.80, *p* < 0.001, *I*^2^ 66%) and 0.77 for women (95% CI 0.67–0.89, *p* < 0.001, *I*^2^ 62%) (Figure 2A,B). To explore substantial heterogeneity and identify gender-related differences in OS according to the mechanism of action of the specific ICIs tested in the trials, we separately analyzed studies investigating anti-PD-1 or anti-PDL-1 agents and those investigating anti-CTLA-4 agents. The combination of anti-CTLA-4 plus anti-PD-1/PD-L1 was investigated in only 2 trials [49,50] and data were not adequate to perform meta-analysis. The use of anti-PD-1/PDL-1 resulted in better outcome both in men and women (HR 0.69, 95% CI 0.62–0.78, *p* < 0.001 and HR 0.73, 95% CI 0.60–0.89, *p* = 0.002, respectively), even when the NSCLC subgroup (including 6 studies) was separately considered (HR 0.73, 95% CI 0.63–0.84, *p* < 0.001 for men and HR 0.66, 95% CI 0.48–0.91, *p* = 0.011 for women) (Figure 3A,B). In contrast, the anti-CTLA-4 treatment was effective in men (HR 0.77, 95% CI 0.63–0.94, *p* = 0.012) (Figure 4A), but did not reach significance in women (HR 0.89, 95% CI 0.76–1.05, *p* = 0.162) (Figure 4B). However, anti-CTLA-4 resulted in a similar benefit in men and women when the analysis was restricted to the 4 studies on melanoma (HR 0.67, 95% CI 0.50–0.90, *p* = 0.008 and HR 0.80, 95% CI 0.68–0.94, *p* = 0.006, respectively) (Figure 4A,B). 

Based on the visual inspection of the funnel plots, we found no suggestion of publication bias (Figure S1, supplementary material). 

### 3.4. Effect of Gender on Progression Free Survival

**Figure 5:**
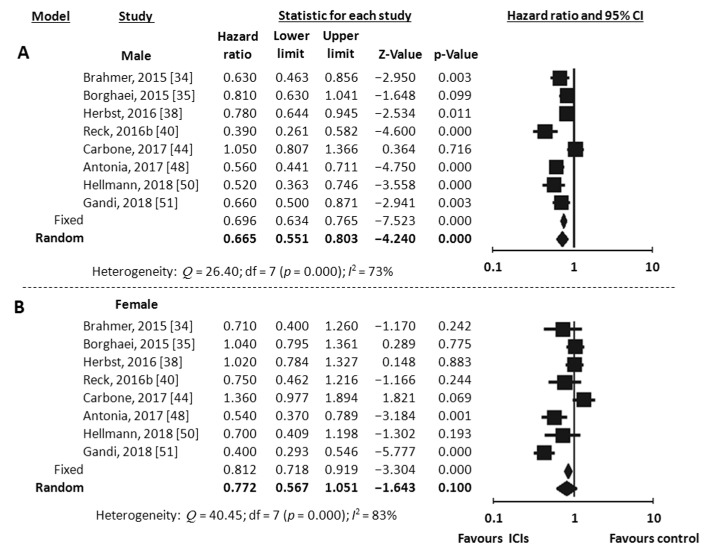
Meta-analysis results for PFS with immune checkpoint inhibitors (ICIs). (**A**), male; (**B**), female.

Eight RCTs reported data on PFS according to gender [34,35,38,40,44,48,50,51]. All of them were trials conducted in patients affected by NSCLC. All, but one [50], investigated anti-PD-1/PDL-1 agents. Meta-analysis using the random-effects model revealed a significant improvement in PFS in men (HR 0.67, 95% CI 0.55–0.80, *p* < 0.001, *I*^2^ 73%) (Figure 5A), but not in women (HR 0.77, 95% CI 0.57–1.05, *p* = 0.100, *I*^2^ 63%) (Figure 5B). 

## 4. Discussion

Gender-related differences of the immune response may translate into differences in the efficacy of ICIs in men and women. Given the lack of clinical trials that considered the potential role of gender in affecting the efficacy of ICIs, we performed a systematic review and meta-analysis of phase III RCTs made available thus far to address this issue. We analyzed OS data from 18 studies including 11,318 patients, and PFS data from 8 studies including 3746 patients. 

We found that both genders gained OS advantage from treatment with ICIs compared to their counterparts, with a tendency towards more favorable outcome in men who reached lower HR. However, a significant improvement in PFS emerged exclusively in men. A benefit in OS was observed in both genders when the analysis was focused on anti-PD-1/PDL-1. Interestingly, men but not women showed a significant better OS when treated with anti-CTLA-4, with the exception of melanoma patients, with women showing a benefit as well. Taken together, our data indicate that ICIs are more effective in men than in women, especially when anti-CTLA-4 agents are considered. 

Three different meta-analyses have been recently published with the aim of assessing gender-related differences in ICI efficacy [25,26,27]. 

In the meta-analysis by Botticelli et al. [25], data from 11 phase II/III trials were included and stratified according to the target of the studied drug. Thus, subgroup analyses were performed to investigate OS with anti-CTLA-4 in 2 trials (1178 patients), OS with anti-PD-1 in 6 trials (3792 patients), and PFS with anti-PD-1 in 6 trials (3274 patients). The study reported a statistical significant improvement of PFS, but not of OS, in men compared to women when treated with anti-PD-1 versus control. Moreover, a more favorable OS was associated with anti-CTLA4 treatment in males, although at a not statistically significant extent. 

The second study by Wu et al. [26] had a similar design and included almost the same phase II/III trials. A meta-analysis was performed to investigate OS in 9 trials (5251 patients) and PFS in 4 trials (2150 patients). Subgroup analyses by type of cancer (melanoma vs. NSCLC) and type of ICI (CTLA-4 inhibitors vs. PD-1 inhibitors) were also performed. Compared to controls, both PFS and OS resulted significantly improved by treatment with ICIs in both genders, but men showed lower HR than women, particularly when treated with anti-CTLA-4 in advanced melanoma. 

In the third study Conforti et al. [27] directly tested the difference in OS HR between men and women treated with ICIs. A meta-analysis was carried out by including data from 20 phase II/III trials, which overall enrolled 11,351 patients. Compared to the control groups, both men and women treated with ICIs showed a reduced risk of death, but men had a significant lower HR. In a subgroup analyses of 6 trials women did not obtain benefit from anti-CTLA-4 agents compared to control, consistent with our results. However, given that the study was aimed at comparing the pooled HR in men vs. the pooled HR in women, the authors emphasized the increased relative benefit in male patients, but did not focus on the fact that anti-CTLA-4 therapy was not superior to control in female. Herein we try to discuss this result. 

All these three studies showed that anti-CTLA-4 treatment tends to improve survival in men, but not in women. In our study, as in the Conforti’s one, this evidence reached the pre-set threshold for statistical significance probably because of the higher number of trials included in the analysis, also encompassing those published over the past year. This may have helped increase the statistical power of our meta-analysis. From a methodological standpoint, compared with the aforementioned studies, we included only phase III RCTs because they are sufficiently powered to detect differences between two groups of treatment, in this case between ICI treatment (experimental arm) and standard therapy or placebo (control arm). Moreover, compared to phase II studies, phase III trials ensure longer follow-up and a higher number of events. Most of the trials included in the Conforti’s study have been considered in our meta-analysis, excluding 2 phase II studies [52,53], 1 study presented as abstract but never published [54], and 1 study that used ICI also in the control arm [55]. In our opinion the latter study is useful to compare two different ICIs, but cannot be informative of the effect of ICIs on survival compared to standard therapy and cannot be used to investigate gender differences. In addition, as mentioned in the introduction, we included 2 more recent phase III studies that were not available at the time of Conforti’s publication [49,51]. Overall, our meta-analysis differs from Conforti’s one by 6 trials, 2 new trials added and 4 not included. 

The emerged evidence for the lack of efficacy of anti-CTLA-4 in women invites critical interpretation. The renowned gender-related differences in immune response [13] and the different mechanisms of action of anti-CTLA-4 and anti-PD-1/PDL-1 [56,57] may help provide a biologic rationale to this finding. 

CTLA-4 is expressed on T lymphocytes and, by binding B7 receptors on antigen-presenting cells (APCs), determines inhibition of T-cell activation at the priming phase of the immune response, when a naïve T lymphocyte recognizes tumor antigens for the first time [56]. Therefore, anti-CTLA-4 antibodies can re-activate suppressed T lymphocytes, stimulating their proliferation and triggering humoral and cytotoxic anti-tumor response. It is widely accepted that this early phase of immune system activation is stronger in women than in men, given that the former have more effective APCs and higher number of CD4 + T cell [13]. It is conceivable that a tumor growing in a human female organism, in order to progress and overcome the host proficient immune response, must select cellular clones with low immunogenic potential, i.e., clones that do not display antigens able to elicit an anti-tumor response. Therefore, in this female-biased immune scenario, we may hypothesize that the lack of T cell activation rather than CTLA-4-mediated T cell inhibition is responsible for tumor escape from immune surveillance. Alternatively, it is also possible that female T-cell suppression is driven by distinct cellular mechanisms which do not include CTLA-4. As a consequence, anti-CTLA-4 therapy fails to revert immune response in women. Moreover, it has been reported that CTLA-4 is expressed in Treg cells, a lymphocyte population with immunosuppressive effects, and that anti-CTLA-4 agents can restore immune competence partly by depleting Treg cells or abrogating their function [58,59]. As women have a lower Treg count than men, they may receive minor benefit from anti-CTLA-4 therapy. 

Unexpectedly, this result was not duplicated in female patients affected by melanoma (4 trials). In this subgroup women, as well as men, received survival advantage by anti-CTLA-4 compared to control. However, our study had not a sufficient statistical power to conclude that the lack of benefit from anti-CTLA-4 in females, in the overall population, was due to the presence in the analysis of tumor types different from melanoma that do not respond to this therapy. In fact, only two trials explored this issue and were not suitable to be considered separately for meta-analysis. 

We can hypothesize that tumor- and/or patient-specific factors other than gender may influence response to anti-CTLA-4 agents in melanoma. In particular, melanoma is known to be a tumor with a very high mutational burden (0.5 to >100 mutations per megabase) [60] and with a high propensity to generate neoantigens that are recognized by the immune system as nonself [61]. The genetic basis for clinical response to anti-CTLA-4 in melanoma have been recently elucidated [61]. We could speculate that melanoma cells are by themselves able to elicit a strong antitumor immune response potentially able to destroy the tumor if the immunosuppressive effect of CTLA-4 expression on T cell does not occur. In this scenario, both men and women receive a benefit from CTLA-4 blockade in melanoma. 

PD-1 is also expressed in T lymphocytes and, similarly to CTLA-4, inhibits T-cell activation by binding PD-L1 and PD-L2 on APCs in the priming phase of the immune response [56]. Notably, PDL-1 and PDL-2 are also expressed in tumor cells and inhibit the cytotoxic activity of CD8+ T lymphocytes against tumor. Thus, the inhibition of PD-1 interaction with its ligands, using anti-PD-1 or anti-PDL-1 antibodies, can re-activate effector CD8+ T cell to kill tumor cells. In light of our results, this peripheral immune mechanism seems to be resumed by anti PD-1/PD-L1 in both genders. Men have a higher number of CD8+ T lymphocytes, but these cells are functionally more active in women [13]. Recent studies have shown that tumor cells of NSCLC express significant higher levels of PD-L1 in male compared to female patients [62]. For this reason, anti-PD1/PDL-1 therapies may be more effective in men. Consistently, our study showed better OS and a significantly improved PFS in men compared to women. 

## 5. Conclusions

The results of our systematic review and meta-analysis indicate that treatment with ICIs improves prognosis in patients affected by different types of cancer, but with a higher benefit for men compared to women, especially when anti-CTLA-4 agents are used. Prospective clinical trials stratified by gender in the randomization process may significantly add to a deeper comprehension of the role of gender in the anti-tumor activity of ICIs. A better understanding of the molecular mechanisms involved in the tumor immune escape would also help identify predictors of response/resistance to ICIs, differently expressed in men and women. 

## Figures and Tables

**Table 1:** Characteristics of the RCTs included in the meta-analysis.

Clinical Trial	Neoplasm (Target)	Treatment	No. Patients		Sex difference HR (95% CI)	
			Male	Female	OS	PFS
Hodi, F.S., 2010 [31]	Melanoma (CTLA-4)	Ipilimumab + Gp100	247	156	Male: 0.66 (0.50–0.87) Female. 0.72 (0.52–0.99	
		Ipilimumab	81	56	Male: 0.54 (0.37–0.77) Female: 0.81 (0.55–1.20)	
		Gp100	73	63		
Robert, C., 2011 [32]	Melanoma (CTLA-4)	Ipilimumab + Dacarbazina	152	98	Male: 0.70 (0.55–0.89) Female: 0.86 (0.63–1.17)	
		Placebo + Dacarbazina	149	103		
Ribas, A., 2013 [33]	Melanoma (CTLA-4)	Tremelimumab	190	138	Male: 0.93 (0.78–1.10) Female: 0.81 (0.61–1.07)	
		Chemotherapy	182	145		
Brahmer, J., 2015 [34]	Squamous NSCLC (PD-1/PDL-1)	Nivolumab	111	24	Male: 0.57 (0.41–0.78) Female: 0.67 (0.36–1.25)	Male: 0.63 (0.63–1.04) Female: 0.71 (0.40–1.26)
		Docetaxel	97	40		
Borghaei, H., 2015 [35]	Nonsquamous NSCLC (PD-1/PDL-1)	Nivolumab	151	141	Male: 0.73 (0.56–0.96) Female: 0.78 (0.58–1.04)	Male: 0.81 (0.56–0.96) Female: 1.04 (0.80–1.37)
		Docetaxel	168	122		
Motzer, R.J., 2015 [36]	Renal-Cell Carcinoma (PD-1/PDL-1)	Nivolumab	315	95	Male: 0.73 (0.58–0.92) Female: 0.84 (0.57–1.24)	
		Everolimus	304	107		
Robert, C., 2015 [37]	Melanoma (PD-1/PDL-1)	Nivolumab	121	89	Male: 0.34 (0.22–0.54) Female: 0.56 (0.33–0.95)	
		Dacarbazina	125	83		
Herbst, R.S., 2016 [38]	NSCLC (PD-1/PDL-1)	Pembrolizumab	425	266	Male: 0.65 (0.52–0.81) Female: 0.69 (0.51–0.94)	Male: 0.78 (0.64–0.94) Female: 1.02 (0.78–1.32)
		Docetaxel	209	134		
Reck, M., 2016a [39]	Small-Cell Lung Cancer (CTLA-4)	Ipilimumab + Chemotherapy	317	161	Male: 1.07 (0.89–1.28) Female: 1.06 (0.81–1.37)	
		Placebo + Chemotherapy	326	150		
Reck, M., 2016b [40]	PD-L1-positive NSCLC (PD-1/PDL-1)	Pembrolizumab	92	62		Male: 0.39 (0.26–0.58) Female: 0.75 (0.46–1.21)
		Chemotherapy	95	56		
Ferris, R.L., 2016 [41]	Head and Neck cancer (PD-1/PDL-1)	Nivolumb	197	43	Male: 0.65 (0.48–0.88) Female: 0.93 (0.47–1.85)	
		Chemotherapy	103	18		
Rittmeyer A, 2017 [42]	NSCLC (PD-1/PDL-1)	Atezolizumab	261	164	Male: 0.79 (0.64–0.97) Female: 0.64 (0.49–0.85)	
		Docetaxel	259	166		
Bellmunt, J., 2017 [43]	Urothelial Carcinoma (PD-1/PDL-1)	Pembrolizumab	200	70	Male: 0.73 (0.56–0.94) Female: 0.78 (0.49–1.24)	
		Chemotherapy	202	70		
Carbone, D.P., 2017 [44]	NSCLC (PD-1/PDL-1)	Nivolumb	184	87	Male: 0.97 (0.74–1.26) Female: 1.15 (0.79–1.66)	Male: 1.05 (0.81–1.37) Female: 1.36 (0.98–1.90)
		Chemotherapy	148	122		
Kang, Y.K., 2017 [45]	Gastric cancer (PD-1/PDL-1)	Nivolumab	229	101	Male: 0.59 (0.46–0.75) Female: 0.83 (0.56–1.23)	
		Placebo	119	44		
Govindan, R., 2017 [46]	Squamous NSCLC (CTLA-4)	Ipilimumab + Chemotherapy	326	62	Male: 0.85 (0.71–1.02) Female: 1.33 (0.84–2.15)	
		Placebo + Chemotherapy	309	52		
Larkin, J., 2017 [47]	Melanoma (PD-1/PDL-1)	Nivolumab	176	96	Male: 0.85 (0.62–1.17) Female:1.07 (0.69–1.65)	
		Investigator’s choice	85	48		
Antonia, S.J., 2017 [48]	NSCLC (PD-1/PDL-1)	Durvalumab	334	142		Male: 0.54 (0.41–0.71) Female: 0.54 (0.37–0.79)
		Placebo	166	71		
Motzer, R.J., 2018 [49]	Renal-Cell Carcinoma (CTLA-4 + PD-1/PDL-1)	Ipilimumab + Nivolumab	314	111	Male: 0.71 (0.55–0.92) Female: 0.52 (0.34–0.78)	
		Sunitinib	301	121		
Hellmann, M.D., 2018 [50]	NSCLC (CTLA-4 + PD-1/PDL-1)	Ipilimumab + Nivolumab	98	41		Male: 0.42 (0.36–0.74) Female: 0.70 (0.41–1.20)
		Chemotherapy	106	54		
Gandi, L., 2018 [51]	Nonsquamous NSCLC (PD-1/PDL-1)	Pembrolizumab	254	156	Male: 0.70 (0.50–0.99) Female: 0.29 (0.19–0.44)	Male: 0.66 (0.50–0.87) Female: 0.40 (0.29–0.54)
		Chemotherapy	109	97		

## Supplementary Materials

The following are available online at http://www.mdpi.com/2077-0383/7/12/542/s1, Table S1: Risk of Bias (Low/unclear/High) for the selected studies, Figure S1: Funnel plot of Standard Error by Log Hazard Ratio of selected studies included in OS analysis; A, male; B, female.
