# Supplementary material for: Effect of Gender on the Outcome of Patients Receiving Immune Checkpoint Inhibitors for Advanced Cancer: A Systematic Review and Meta-Analysis of Phase III Randomized Clinical Trials
Source: J Clin Med. 2018 Dec 12;7(12):542. doi: 10.3390/jcm7120542 (PMC6306894; doi:10.3390/jcm7120542)
Supplement: Supplementary file 1 [file jcm-07-00542-s001.zip › Table 1S.docx]

**Table 1S: Risk of Bias (Low/unclear/High) for the selected studies.**

| **Study bias** | **Risk of bias** | **Quoting and Notes** |
| --- | --- | --- |
|  |  |  |
| **Hodi 2010 [31]** |  |  |
| Randomization sequence generation (Sequ Gen) (Selection bias) | Unclear | No details reported |
| Allocation concealment (All Conc)  (Selection Bias) | Unclear | No details reported |
| Blinding of pts and personnel  (Performance Bias) | Low Risk | Quoting “Double Blind” |
| Blinding of outcome assessors  (Detection Bias) | Unclear | No details reported |
| Incomplete Outcome Data  (Attrition) | Unclear | Percentage (%) of pts lost to follow up (Fup) and exit strategies for missing data are not clearly addressed |
| Selective Reporting | Low risk | Trial registered, the same outcomes are addressed across the different manuscript (Ms) sections |
| Other Bias | Low risk | None suspected |
| ITT | Yes | Quoting “Efficacy analyses were performed on the intention-to-treat population” |
|  |  |  |
| **Robert C 2011 [32]** |  |  |
| Selection Bias (Sequ Gen) | Unclear | No details reported |
| Selection Bias (All Conc) | Unclear | No details reported |
| Performance Bias | Low Risk | Quoting “Double Blind |
| Detection Bias | Unclear | No details reported |
| Attrition Bias | Unclear | Particpiants lost to Fup and exit strategies for missing data are not clearly addressed |
| Selective Reporting | Low risk | Trial registered, same outcomes across manuscript sections |
| Other Bias | Low risk | None suspected |
| ITT | Yes | Quoting “Efficacy analyses were performed on the intention-to-treat population” |
|  |  |  |
| **Ribas 2013 [33]** |  |  |
| Selection Bias (Sequ Gen) | Unclear | No details reported |
| Selection Bias (All Conc) | High risk | Allocation unmasked |
| Performance Bias | High risk | Open-label |
| Detection Bias | Unclear | No details reported |
| Attrition Bias | Unclear | Participants lost to Fup and exit strategies for missing data are not clearly addressed |
| Selective Reporting | Low risk | Study registered, consistency in outcome reporting across the Ms sections |
| Other Bias | Low risk | None suspected |
| ITT | Yes | Based on the numbers reported in tables and figures |
|  |  |  |
| **Brahamer 2015 [34]** |  |  |
| Selection Bias (Sequ Gen) | Unclear | No details provided |
| Selection Bias (All Conc) | Unclear | No details provided |
| Performance Bias | High risk | Open-label |
| Detection Bias | Unclear | No details provided |
| Attrition Bias | Unclear | Participants lost to Fup and exit strategies for missing data are not clearly addressed |
| Selective Reporting | Low risk | Trial registered, consistency in outcome reporting across the different sections of the Ms |
| Other Bias | Low risk | None suspected |
| ITT | Yes | Quoting “Demographic and efficacy analyses included all the patients who underwent randomization (intention-to-treat population)” |
|  |  |  |
| **Borghaei 2015 [35]** |  |  |
| Selection Bias (Sequ Gen) | Unclear | No details reported |
| Selection Bias (All Conc) | Unclear | No details reported |
| Performance Bias | Low | Placebo-controlled |
| Detection Bias | Unclear | No details reported |
| Attrition Bias | Low | Quoting “All randomized participants were followed up for survival, unless they had withdrawn consent for survival follow up. Survival info were obtained through a search of publicly available sources for patients who withdrew consent or were lost to follow up” |
| Selective Reporting | Low | Study registered, consistency in outcomes reporting across the different sections of the Ms |
| Other Bias | Low | None suspected |
| ITT | Yes | Quoting “Demographic and efficacy analyses included all the patients who underwent randomization.” |
|  |  |  |
| **Motzer 2015** [36] | | |
| Selection Bias (Sequ Gen) | Unclear | No details reported |
| Selection Bias (All Conc) | High risk | Allocation unmasked |
| Performance Bias | High risk | Open-label |
| Detection Bias | Unclear | No details reported |
| Attrition Bias | Unclear | No details reported |
| Selective Reporting | Low | Study registered, consistency in outcome reporting across the different sections of the Ms |
| Other Bias | None suspected | None suspected |
| ITT | Yes | Based on figure 1 and 2 |
|  |  |  |
| **Robert C, 2015 [37]** |  |  |
| Selection Bias (Sequ Gen) | Unclear | No details reported |
| Selection Bias (All Conc) | High | Allocation unmasked |
| Performance Bias | High | Open label |
| Detection Bias | Unclear | No details reported |
| Attrition Bias | Unclear | No details reported |
| Selective Reporting | Low risk | Study registered, consistency in outcome reporting across the different sections of the Ms |
| Other Bias | Low risk | None suspected |
| ITT | Yes | Quoting “Efficacy was assessed in the intention-to-treat population” |
| Early stop | Yes | Trial stopped for efficacy |
|  |  |  |
| **Herbst 2016 [38]** |  |  |
| Selection Bias (Sequ Gen) | Low | Quoting “Patients were randomly assigned (1:1:1) with a central interactive voice-response system” and “The allocation schedule was generated by the system vendor using a computerized randomized list generator.” |
| Selection Bias (All Conc) | High | Allocation unmasked |
| Performance Bias | High | Open-label |
| Detection Bias | Unclear | No details reported |
| Attrition Bias | Low | Quoting “For overall survival, data for patients who were alive or lost to follow-up were censored at the time of last confirmed contact.  For progression-free survival, data for patients who had not progressed or were lost to follow-up were censored at the time of last tumour assessment.” |
| Selective Reporting | Low | Study registered, consistency in outcome reporting across the different sections of the Ms  , |
| Other Bias | Low | None suspected |
| ITT | High | Quoting “After one patient was allocated to and received pembrolizumab 2 mg/kg, it was found that their pre-baseline scans were not compliant with the protocol. The patient was permitted to remain on treatment and was included in the safety analysis population, but because it would not be possible to adequately assess tumour response, the patient was excluded from the efficacy analysis population.” |
|  |  |  |
| **Reck M, 2016a [39]** |  |  |
| Selection Bias (Sequ Gen) | Low | Quoting “Patients were randomly assigned at a ratio of one to one to receive ipilimumab 10 mg/kg intravenously (IV) or placebo using an interactive voice response system with a stratified, permuted, blockrandomization method” |
| Selection Bias (All Conc) | Low | Placebo-controlled trial. |
| Performance Bias | Low | Quoting “Research staff was blinded to treatment assignment.” |
| Detection Bias | Low | Quoting “Research staff was blinded to treatment assignment.” |
| Attrition Bias | High | 4 pts lost to fup in the treatment arm and 1 in the control arm (Consort diagram) |
| Selective Reporting | Low | Study registered, consistency in outcome reporting across the different sections of the Ms |
| Other Bias | Low | None suspected |
| ITT | No | Quoting “Secondary end points included OS in all randomly assigned patients and PFS per modified WHO criteria among patients who received at least  one dose of study therapy” |
|  |  |  |
| **Reck M, 2016b [40]** |  |  |
| Selection Bias (Sequ Gen) | Unclear | No details reported |
| Selection Bias (All Conc) | High | Allocation Unmasked |
| Performance Bias | High | Open label |
| Detection Bias | Low | Quoting “The primary end point, progression-free survival, was assessed by means of blinded, independent, central radiologic review.” |
| Attrition Bias | Low | Quoting “For the analysis of overall survival (and PFS), data for patients who were alive or who were lost to follow-up were censored at the time of the last contact.” |
| Selective Reporting | Low | Study registered, consistency in outcome reporting across the different sections of the Ms |
| Other Bias | Low risk | None suspected |
| ITT | Yes | Quoting “Efficacy was assessed in the intention-to-treat population, which included all patients who underwent randomization” |
|  |  |  |
| **Ferris RL, 2016 [41]** |  |  |
| Selection Bias (Sequ Gen) | Unclear | No details reported |
| Selection Bias (All Conc) | High | Allocation unmasked |
| Performance Bias | High | Open label |
| Detection Bias | Unclear | No details reported |
| Attrition Bias | Unclear | No details reported |
| Selective Reporting | Low risk | Study registered, consistency in outcome reporting across the different sections of the Ms |
| Other Bias | Low risk | None suspected |
| ITT | Yes | Quoting “Analyses of baseline characteristics and efficacy followed the intention-to-treat principle.” |
|  |  |  |
| **Rittmeyer A, 2017 [42]** |  |  |
| Selection Bias (Sequ Gen) | Low | Quoting “Patients were randomly assigned by permuted block randomisation (block size of eight) via an interactive voice or web response system.” |
| Selection Bias (All Conc) | High Risk | Allocation unmasked |
| Performance Bias | High Risk | Open-label |
| Detection Bias | Unclear | No details reported |
| Attrition Bias | Unclear | No details reported |
| Selective Reporting | Low | Study registered, consistency in outcome reporting across the different sections of the Ms |
| Other Bias | Low | None suspected |
| ITT | Yes | Repeatedly mentioned throughout the abstract and main body text. |
|  |  |  |
| **Bellmunt J, 2017 [43]** |  |  |
| Selection Bias (Sequ Gen) | Unclear | No details reported |
| Selection Bias (All Conc) | High | Allocation unmasked |
| Performance Bias | High | Open-label |
| Detection Bias | Unclear | No details reported |
| Attrition Bias | Low | Quoting “Patients who were alive or lost to follow up had their data censored at the time of last contact.” |
| Selective Reporting | Low | Study registered, consistency in outcome reporting across the different sections of the Ms |
| Other Bias | Low | None suspected |
| ITT | Yes | Quoting “Efficacy was assessed in the intention-to-treat population, which included all the patients who were assigned to a treatment group.” |
|  |  |  |
| **Carbone DP, 2017 [44]** |  |  |
| Selection Bias (Sequ Gen) | Unclear | No details reported |
| Selection Bias (All Conc) | High | Allocation unmasked |
| Performance Bias | High | Open label |
| Detection Bias | Low (with reserve) | Quoting “Progression-free survival was assessed by blinded independent central review, among patients with a PD-L1 expression level of 5% or more.”, that is, the blinded assessment was restricted to a subgroup only. |
| Attrition Bias | Unclear | No details reported |
| Selective Reporting | Low | Study registered, consistency in outcome reporting across the different sections of the Ms |
| Other Bias | Low | None suspected |
| ITT | No | Quoting “Overall, 530 patients (98% of all the patients who had undergone randomization) received treatment.” And “The primary efficacy analysis population (423 patients with a PD-L1 expression level of ≥5%) constituted 78% of all the patients who had undergone randomization.” |
|  |  |  |
| **Kang YK, 2017 [45]** |  |  |
| Selection Bias (Sequ Gen) | Low | Quoting “Patients were randomly assigned (2:1) using an interactive web response system” |
| Selection Bias (All Conc) | Low | Quoting “A non-masked  pharmacist prepared nivolumab and the placebo (saline) according to a written protocol to maintain blinding of treatment assignment; no nivolumab-matching placebo  was supplied. The supply and management of nivolumab  were overseen by additional non-masked monitors. Both  nivolumab and placebo solutions were colourless and could not be visually distinguished.” |
| Performance Bias | Low | Double-blind, placebo controlled trial |
| Detection Bias | Unclear | Not specifically reported whether outcome assessors were blinded. Quoting “Patients and investigators were blinded” |
| Attrition Bias | Unclear | No details reported |
| Selective Reporting | Low | Study registered, consistency in outcome reporting across the different sections of the Ms |
| Other Bias | Low | None suspected |
| ITT | Yes | Quoting “The primary endpoint was overall survival in the intention-to-treat population.” |
|  |  |  |
| **Govindan R, 2017 [46]** |  |  |
| Selection Bias (Sequ Gen) | Unclear | No details provided |
| Selection Bias (All Conc) | Low | Quoting “This randomized, double-blind, phase III study investigated. […]” |
| Performance Bias | Low | Quoting “This randomized, double-blind, phase III study investigated. […]” |
| Detection Bias | Unclear | No details provided |
| Attrition Bias | Unclear | Only early withdrawals clearly mentioned (CONSORT diagram) |
| Selective Reporting | Low | Study registered, consistency in outcome reporting across the different sections of the Ms |
| Other Bias | Low | None suspected |
| ITT | No | Quoting “The primary end point was OS among all randomly assigned patients who received at least one dose of blinded therapy (modified intent-to-treat [mITT] population)” |
|  |  |  |
| **Larkin J, 2017 [47]** |  |  |
| Selection Bias (Sequ Gen) | Unclear | No details reported |
| Selection Bias (All Conc) | High | Allocation unmasked |
| Performance Bias | High | Open-label |
| Detection Bias | Unclear | No details reported |
| Attrition Bias | Unclear | Patients lost to Fup by study arm is reported (23 vs 1, in the control and intervemtion arm). No further details are reported. |
| Selective Reporting | Low | Study registered, consistency in outcome reporting across the different sections of the Ms |
| Other Bias | Low risk | None suspected |
| ITT | Yes | Quoting “Efficacy end points were based on the intent-to-treat population” |
|  |  |  |
| **Antonia SL, 2017 [48]** |  |  |
| Selection Bias (Sequ Gen) | Unclear | No details reported |
| Selection Bias (All Conc) | Unclear | No details reported |
| Performance Bias | Low | Placebo-controlled |
| Detection Bias | Low | Quoting “Endpoints assessed by means of blinded independent central review” |
| Attrition Bias | Low | Quoting “Sensitivity analyses included assessment of evaluation bias, evaluation-time bias, and attrition bias in the determination of disease progression and adjustment for various covariates in the estimation of the hazard ratio for disease progression or death.” |
| Selective Reporting | Low | Study registered, consistency in outcome reporting across the different sections of the Ms |
| Other Bias | Low | None suspected |
| ITT | Yes | Quoting “Efficacy was assessed in the intention-to-treat population” |
|  |  |  |
| **Motzer RJ, 2018 [49]** |  |  |
| Selection Bias (Sequ Gen) | Unclear | The methods used to generate the randomization sequence are not clearly reported, although the use of blocks and stratification may be in support of a centralized, computer assisted randomization. Quoting “Randomization (in a 1:1 ratio) was performed with a block size of 4 with stratification” |
| Selection Bias (All Conc) | High | Allocation unmasked |
| Performance Bias | High | Open-label |
| Detection Bias | Low | Independent review for the study endpoints |
| Attrition Bias | Unclear | No details reported |
| Selective Reporting | Low | Study registered, consistency in outcome reporting across the different sections of the Ms |
| Other Bias | Low | None suspected |
| ITT | Yes | Quoting “In the intention-to-treat population” |
|  |  |  |
| **Hellmann MD, 2018 [50]** |  |  |
| Selection Bias (Sequ Gen) | Unclear | No details reported |
| Selection Bias (All Conc) | High | Allocation unmasked |
| Performance Bias | High | Open-label |
| Detection Bias | Low | Quoting “PFS assessed by blinded independent central review” |
| Attrition Bias | Unclear | No details reported |
| Selective Reporting | Low risk | Study registered, consistency in outcome reporting across the different sections of the Ms |
| Other Bias | Low risk | None suspected |
| ITT | Yes | Quoting “Among all randomly assigned patients (irrespective of tumor mutational burden or PD-L1 expression level), the 1-year progression-free survival rate was higher with nivolumab plus ipilimumab than with chemotherapy.” |
| **Gandi L, 2018 [51]** |  |  |
| Selection Bias (Sequ Gen) | Low | Quoting “Randomization was performed by means of  an integrated interactive voice-response and Webresponse  system (i.e., treatment assignments could be provided by following a series of prompts on a touch-tone phone or by following the same  prompts in a Web-based portal)” |
| Selection Bias (All Conc) | Unclear | No details reported |
| Performance Bias | Low | Double-blind trial |
| Detection Bias | Low | Quoting “The primary end points were overall survival and progression-free survival, as assessed by blinded, independent central radiologic review.” |
| Attrition Bias | High | Quoting “Data for patients who were alive or lost to follow-up were censored for overall survival at the time they were last known to be alive” |
| Selective Reporting | Low | Study registered, consistency in outcome reporting across the different sections of the Ms |
| Other Bias | Low | None suspected |
| ITT | Yes | Quoting “Efficacy was assessed in the intention-to-treat population, which included all the patients who had undergone randomization”. |
