# Supplementary figures and images for: Effect of Gender on the Outcome of Patients Receiving Immune Checkpoint Inhibitors for Advanced Cancer: A Systematic Review and Meta-Analysis of Phase III Randomized Clinical Trials
Source: J Clin Med. 2018 Dec 12;7(12):542. doi: 10.3390/jcm7120542 (PMC6306894; doi:10.3390/jcm7120542)

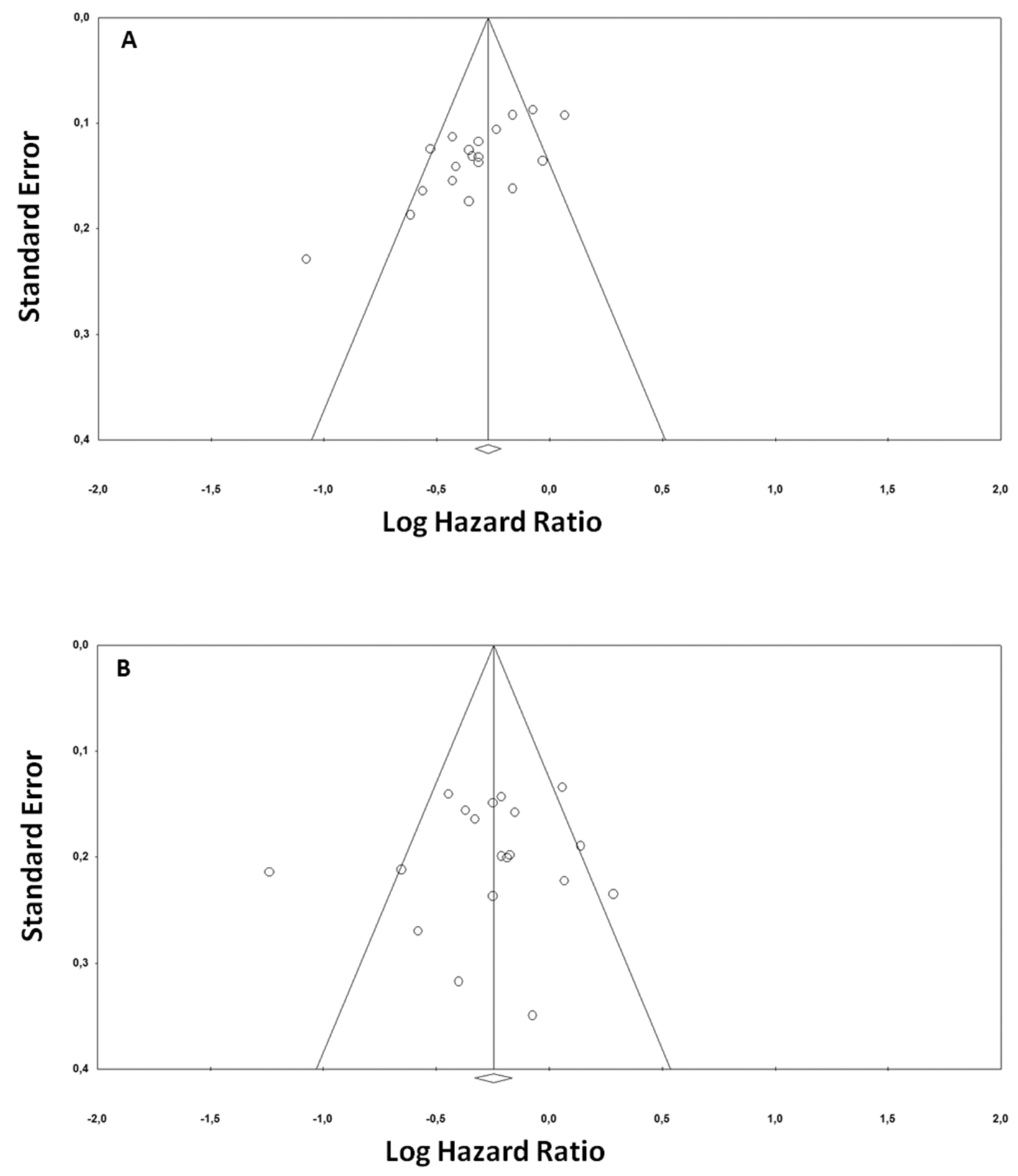

Supplement: Supplementary file 1 [file jcm-07-00542-s001.zip › figure1S.tif]
